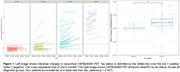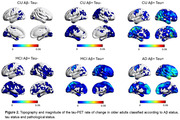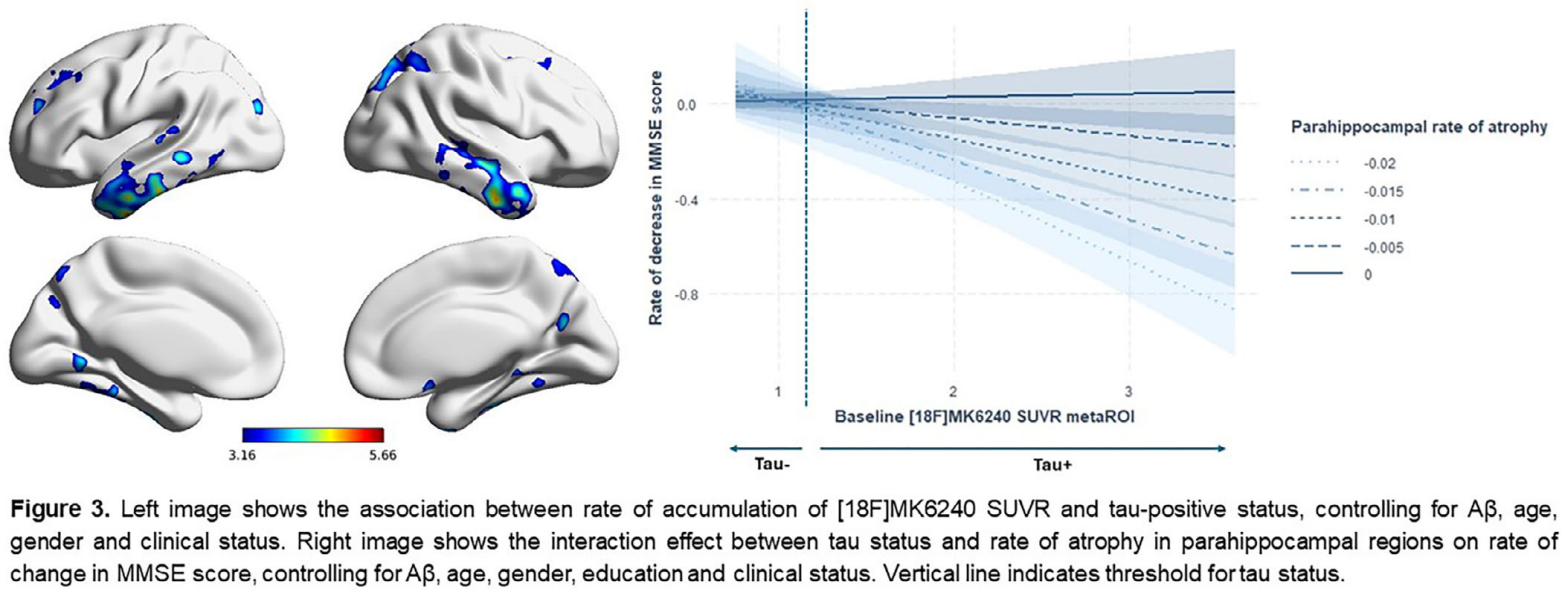# Synergic effect of tau tangles and cortical thinning on cognitive decline in aging and Alzheimer’s disease

**DOI:** 10.1002/alz.085973

**Published:** 2025-01-09

**Authors:** Lydia Trudel, Joseph Therriault, Nesrine Rahmouni, Etienne Aumont, Yi‐Ting Wang, Stijn Servaes, Arthur C. Macedo, Tevy Chan, Jaime Fernandez Arias, Brandon J Hall, Seyyed Ali Hosseini, Kely Monica Quispialaya Socualaya, Sulantha Mathotaarachchi, Jenna Stevenson, Firoza Z Lussier, Serge Gauthier, Tharick A. Pascoal, Pedro Rosa‐Neto

**Affiliations:** ^1^ McGill University, Montreal, QC Canada; ^2^ Translational Neuroimaging Laboratory, The McGill University Research Centre for Studies in Aging, Montréal, QC Canada; ^3^ Université du Québec à Montréal, Montréal, QC Canada; ^4^ University of Pittsburgh, Pittsburgh, PA USA; ^5^ Department of Neurology and Neurosurgery, and Department of Psychiatry, McGill Centre for Studies in Aging, McGill University, Montreal, QC Canada

## Abstract

**Background:**

Tau burden has been found to be involved in brain atrophy during aging, especially in regions such as the parahippocampal gyrus. However, how tau levels at baseline are associated with trajectories of tau accumulation, cortical thinning and cognitive impairment remains poorly understood. The goal of this study was to assess tau rate of change in patients between baseline Tau+ and Tau‐ patients. We also aimed to determine if tau status at baseline or rate of accumulation of tau interacted with the rate of cortical thinning of the parahippocampal regions, leading to an increased rate of change in cognition.

**Method:**

We assessed 133 participants from the TRIAD cohort (mean age = 68.5 ± 8.9, 85 females) with at least two visits with tau‐PET using [^18^F]MK6240 and cortical thickness extracted using FreeSurfer 7. Average time between visits was 25.6 months. Patients were classified according to clinical status, Amyloid‐β status and tau status at baseline (CU AB‐Tau‐, CU AB+Tau‐, CU AB+Tau+, MCI AB+Tau‐, MCI AB+Tau+, AD AB+Tau+). The tau‐PET rate of change was calculated for each participant, voxelwise and with temporal meta‐ROI. The MMSE rate of change was calculated to quantify cognitive decline over follow‐up period. We assessed the voxelwise relationship between baseline tau status and tau‐PET rate of change.

**Results:**

We found a voxelwise positive association between rate of accumulation of tau and tau status at baseline in both anterior temporal lobes. Furthermore, we found an interaction effect between tau‐PET at baseline and parahippocampal rate of atrophy on MMSE rate of change (standardized β = 0.48, p < 0.0001).

**Conclusion:**

Our results suggest that tau status at baseline might impact the rate of accumulation of tau, and that higher baseline levels of tau interact with cortical thinning of parahippocampal regions, leading to faster rates of cognitive decline. Our results emphasize the importance of preventing tau burden to prevent faster pace of cognitive decline during the disease’s trajectory.